# Lectins as Biomarkers of IC/BPS Disease: A Comparative Study of Glycosylation Patterns in Human Pathologic Urothelium and IC/BPS Experimental Models

**DOI:** 10.3390/diagnostics12051078

**Published:** 2022-04-25

**Authors:** Dominika Peskar, Tadeja Kuret, Jera Jeruc, Andreja Erman

**Affiliations:** 1 Faculty of Medicine, Institute of Cell Biology, University of Ljubljana, 1000 Ljubljana, Slovenia; dominika.peskar@mf.uni-lj.si (D.P.); tadeja.kuret@mf.uni-lj.si (T.K.); 2 Faculty of Medicine, Institute of Pathology, University of Ljubljana, 1000 Ljubljana, Slovenia; jera.jeruc@mf.uni-lj.si

**Keywords:** interstitial cystitis/bladder pain syndrome, urothelium, lectin, glycosylation, mouse model, in vitro model

## Abstract

Pathophysiology of interstitial cystitis/bladder pain syndrome (IC/BPS) remains poorly understood, as well as its effective diagnosis and therapy. Studying changes in tissue glycosylation patterns under pathological conditions is a promising way of discovering novel biomarkers and therapeutic targets. The glycobiology of IC/BPS is largely understudied, therefore we compared glycosylation patterns of normal human urothelium with the urothelium of IC/BPS patients using a selection of 10 plant-based lectins with different monosaccharide preferences. We also compared lectin binding to human urothelium with the two most cited experimental models of IC/BPS, specifically, TNFα-treated human urothelial cell line RT4 and cyclophosphamide-induced chronic cystitis in C57BL6/J mice. Furthermore, binding of four of the selected lectins (ConA, DSL, Jacalin and WGA) was evaluated qualitatively by means of fluorescence microscopy, and quantitatively by fluorescence intensity (F.I.) measurements. Our results reveal a significant reduction in F.I. of Jacalin, as well as a prominent change in the WGA labeling pattern in the urothelium of IC/BPS patients, suggesting their potential use as promising additional biomarkers for histopathological diagnosis of IC/BPS. We have also shown that urothelial glycosylation patterns between selected experimental models and patients with IC/BPS are similar enough to offer an adequate platform for preclinical study of IC/BPS glycobiology.

## 1. Introduction

Interstitial cystitis/bladder pain syndrome (IC/BPS) is a debilitating condition with a prevalence of 10.6 cases per 100,000. Women are more commonly affected than men, reaching a ratio of 7:1 [1]. IC/BPS is defined as chronic pelvic pain or pressure perceived to originate from the bladder, accompanied by various urinary symptoms, such as increased frequency of voiding and nocturia, which severely affect patients’ quality of life [2]. The etiology and pathophysiology of the disease remain elusive, resulting in ineffective diagnostic and therapeutic strategies. As clinical symptoms are not IC/BPS-specific, setting the correct diagnosis is often difficult. The 2008 proposal from the International society for the study of BPS (ESSIC) suggests that a patient presenting with chronic pelvic pain lasting at least 6 months, pressure or discomfort related to urinary bladder, accompanied with at least one urinary symptom should first undergo a comprehensive exclusion process to rule out other confounding diseases, such as urinary tract infection, overactive bladder, vulvodynia, and chronic prostatitis in men [3]. Subsequently, the IC/BSP type should be determined based on cystoscopy findings after hydrodistension and pathological findings from biopsy specimens [3]. There are currently no reliable serum or urinary diagnostic biomarkers available for IC/BPS, which is related to insufficient understanding of IC/BPS pathophysiology.

In general, IC/BPS can be categorized into two distinct types based on pathological findings: IC with major pathological changes, such as urothelial erosion and ulceration, referred to as Hunner lesions, and IC without Hunner lesions, with minimal or no pathological changes found in the urothelium [2,4]. Many theories have been proposed to explain its development, most often involving damaged urothelial layer and the associated reduction of the bladder permeability barrier [5]. The urothelium of the mammalian bladder is a specialized epithelium that forms a tight barrier to prevent the exchange of urine and tissue-soluble components. It is composed of basal cells (BC) at the basal lamina, intermediate cells (IC), and superficial cells (SC), also called umbrella cells with a specialized apical plasma membrane reinforced with the transmembrane proteins uroplakins and glycocalyx [6,7]. The glycocalyx, a layer composed of mucin glycosaminoglycans (GAG) and glycoproteins, is thought to play an important role in maintaining the urothelial barrier and was found to be reduced in the bladders of IC/BPS patients [8]. Consequently, intravesical GAG replenishment therapy is one of the currently used strategies for IC/BPS treatment [9]. However, several studies have shown that only trace amounts of GAG could be biochemically isolated from bladders of different species (calf, rabbit), while at the same time high glycoprotein contents were detected, proving that bladder urothelium is highly glycosylated [10,11].

Changes in glycosylation patterns of different cell types have already been shown in various diseases, such as atherosclerosis, inflammatory bowel disease (IBD), and diabetes [12,13,14]. These changes can be detected using lectins—a group of proteins, mostly isolated from plants, with the ability to bind specific sugar moieties [15]. Lectins can interact with mono- and oligosaccharides as well as sugar residues of complex molecules. Their ability to interact with carbohydrates depends on many factors, including their preference for monosaccharides and the structure of their carbohydrate recognition domain (CRD). The specificity of the lectin–carbohydrate interaction in complex molecules can be compared to immune reactions due to the multivalence of lectin binding, which depends on the number and type of subunits that form the lectin, and additional binding sites presented in the structure of branched carbohydrates [15]. The high specificity of lectin–carbohydrate binding has recently led to the development of several lectin-based analytical approaches and their use in the discovery of new biomarkers [15,16]. In the past decades, lectin histochemistry in particular, has been used as a method to distinguish between healthy and pathologically altered tissues. The diagnostic value of lectins has been demonstrated in various intestinal pathologies, such as appendicitis, colonic adenoma, ulcerative colitis, and Chron’s disease [17,18,19,20,21,22,23], as well as cutaneous melanoma [24,25], cholangiocarcinoma [26], and breast cancer [27].

Glycosylation patterns of healthy human urinary bladders have been previously studied using lectin histochemistry, particularly in comparison to bladder cancer tissue [28,29,30,31,32,33]. In addition, glycoconjugate expression has been analyzed in normal bladders of various species, including rabbit [11,34], donkey [35], rat [36,37], and mouse [38], as well as in various urothelial cell lines [39,40,41]. Lectin binding has also been studied in animal models of bladder cancer [31,38] and chronic bacterial cystitis [37], showing specific binding of some lectins to neoplastic bladder tissue, and increased glycosylation of the urothelium in E. coli-induced bladder inflammation, respectively. However, to our knowledge, lectin binding has never been analyzed in in vitro or in vivo models of IC/BPS.

The focus of the present study is the analysis of glycosylation patterns of urothelial cells in normal and IC/BPS human bladder, as well as in in vitro and animal IC/BPS models using a series of FITC-conjugated plant lectins. Our aim was to identify novel biomarkers that could potentially complement the currently used IC/BPS diagnostic methods, and aid in the recognition of lectin-detectable targets for enhanced efficiency of intravesical drug delivery. We also compared lectin binding patterns between the samples of patients with IC/BPS and experimental models of IC/BPS to evaluate the adequacy of preclinically obtained data for extrapolation to glycobiology of IC/BPS in patients. 

## 2. Materials and Methods

### 2.1. Lectins

For initial screening of glycosylation patterns of human and mouse bladder samples and cell cultures, 10 lectins with preference for six basic monosaccharides (fucose, mannose, glucose, galactose, GalNAc, and GlcNAc), and with the affinity for binding to various carbohydrate structures were chosen (Table 1). By selection of these lectins, the detection of a broad spectrum of urothelium-bound carbohydrates was enabled. 

### 2.2. Samples of Patients with IC/BPS

Histological specimens of urinary bladder mucosa from three female patients with Hunner-type IC/BPS and three normal tissue specimens (controls) from male patients were obtained from the archives of the Institute of Pathology, Faculty of Medicine, University of Ljubljana. The biopsy specimens of bladder mucosa were obtained by transurethral resection, fixed in 10% buffered formalin, embedded in paraffin, sliced, and stained with hematoxylin and eosin. The diagnosis of IC/BPS was based on clinical history and physical and laboratory examination with the exclusion of other disorders that could be the cause of the patient’s symptoms. For this study, the original slides were reexamined by a pathologist experienced in urologic histopathology. Histological examination of the IC/BPS slides showed denudation of the urothelium with chronic inflammatory infiltrate of lymphocytes, plasma cells, and mastocytes. Stromal fibrosis and considerable edema were also present in IC/BPS tissue samples. Since normal human urinary bladder specimens cannot be obtained from healthy individuals due to ethical considerations, the morphologically intact bladder mucosa was obtained from patients with bladder cancer undergoing transurethral resection. Among these samples, only parts of tissue with positive immunoreaction against asymmetric unit membrane (AUM) in superficial urothelial cells (the proof of normal, intact urothelium) were considered as normal and used for further analysis.

### 2.3. Animal Model of IC/BPS

Adult C57BL6/J mice (12–16 weeks) of both sexes (10 males and 10 females; *n* = 20), weighing between 20 and 35 g were used for in vivo experiments. Animals were housed in groups of 4–5 in polyacrylamide cages at constant humidity (55%) and temperature (22 °C) with water and food ad libitum, and 12 h/12 h light-dark cycle. All animals were allowed an acclimation period of at least 7 days. Animals were randomly divided into two experimental groups: cyclophosphamide (CYP)-treated group (5 males and 5 females; *n* = 10) and control group (5 males and 5 females; *n* = 10). IC/BPS was induced as previously described [43,44]. Briefly, 80 mg/kg CYP (#C0768, Sigma-Aldrich, Merck, Darmstadt, Germany) diluted in sterile saline was administered i.p. every other day for 8 days (applications on days 0, 2, 4, and 6). The control group received equivalent amounts of sterile saline. Animals were weighed daily and examined for potential signs of pain or physical discomfort. On the eighth day of the experiment, the animals were euthanized by CO_2_-asphyxia. Urinary bladders were excised and fixed in 10% buffered formalin for 24 h, dehydrated, and embedded in paraffin. The 5 μm-thick paraffin sections were used in all following experiments. All animal experiments were conducted in accordance with Administration of the Republic of Slovenia for Food Safety, Veterinary Sector and Plant Protection, permit number U34401-4/2020/10.

### 2.4. In Vitro IC/BPS Model

Human urothelial carcinoma cell line RT4 (HTB-2, ATCC, Manassas, VA, USA) was grown in basal media consisting of equal parts of advanced Dulbecco’s Modified Eagle’s Medium (A-DMEM) (Gibco, Life Technologies, Thermo Fisher Scientific, Waltham, MA, USA) and F12 (HAM) (Sigma-Aldrich, Merck, Darmstadt, Germany) supplemented with 5% fetal bovine serum (FBS; Gibco, Life Technologies, Carlsbad, CA, USA) and 4 mM GlutaMAX (Gibco, Life technologies, Carlsbad, CA, USA) in 75 cm^2^ culture flasks. For the experiments, RT4 cells were seeded on coverslips in 6-well plates (for fluorescence microscopy) or 96-well plates without coverslips (for measuring fluorescence intensity) at a seeding density of 1 × 105 cells/cm^2^ and grown until reaching 80–90% confluence. To mimic a proinflammatory environment, cells were treated with 20 ng/mL human recombinant TNFα (Cayman Chemicals, Ann Arbor, MI, USA) in serum-free basal media for 24 h, as previously described [45,46,47]. Untreated cells grown in serum-free basal media served as controls.

### 2.5. Combined Lectin- and Immuno-Histochemistry (CLIH) and Lectin-Cytochemistry

In the present study, the method of combined lectin- and immuno-histochemistry has been used as described previously [33]. Briefly, paraffin sections of mouse and human bladder samples were deparaffinized and rehydrated in decreasing concentrations of ethanol. After a brief wash in phosphate buffered saline (PBS), the sections were blocked in 3% bovine serum albumin (BSA) in PBS for 1 h at room temperature (RT) and incubated with rabbit polyclonal primary antibodies raised against the AUM, a marker of terminally differentiated SC (diluted 1:10,000 in blocking solution; kind gift from TT Sun, University of New York) overnight at 4 °C. The following day, the sections were incubated in a combined mixture of secondary antibodies (goat-anti rabbit, 1:400, Alexa Fluor 555, Invitrogen, Thermo Fisher, Waltham, MA, USA) and 10 different FITC-conjugated lectins (manufacturers and dilutions are listed in Table 1) in PBS for 1 h at RT. Nuclei were visualized by Höechst (Thermo Fisher Scientific, Carlsbad, CA, USA) staining for 15 min at RT, followed by mounting in Vectashield (Vector Labs, Burlingame, CA, USA). One section from each animal or patient was used for CLIH with each lectin. Sections were examined with the AxioImager.Z1 fluorescence microscope (Zeiss, Jena, Germany) and analyzed semi-quantitatively. Representative microscopic images of labeling with four lectins (ConA, DSL, Jacalin, and WGA), selected for more detailed analysis, were taken under immersion objective (63×/NA 1.40). Negative controls were performed by the omission of lectins in the protocol, while lectin staining of vascular endothelium served as a positive control. 

For lectin-cytochemistry, coverslips with RT4 cells were first fixed with 10% buffered formalin for 15 min at RT, then washed in PBS and blocked in 1% BSA in PBS for 1 h at RT, followed by incubation in dilution of various FITC-conjugated lectins (Table 1) in PBS for 1 h at RT. The coverslips were mounted in Vectashield with DAPI (Vector Labs, Burlingame, CA, USA). The experiment was independently repeated three times. The coverslips were examined with the AxioImager.Z1 fluorescence microscope (Zeiss, Jena, Germany). Representative microscopic images of labeling with ConA, DSL, Jacalin, or WGA were taken at 20× objective magnification.

### 2.6. Lectin Binding Evaluation

The lectin binding to human and mouse urothelium of both control and IC/BPS samples prepared as described above, was first qualitatively and semi-quantitatively assessed. The fluorescence intensity (F.I.) of urothelium-bound FITC-conjugated lectins was evaluated as ++ (very intense), + (intense), +/− (weak), or − (very weak). For cell cultures, the mean F.I. was determined in RT4 cells prepared as described below. The F.I. values were graded as weak (+/−), intense (+), and very intense (++) when a minimum of 4-fold, 12-fold or 25-fold higher values than that of the negative control were obtained, respectively. 

For quantitative analysis of lectin binding to the urothelium, F.I. was measured using AxioVision Rel 4.8 software (Zeiss, Jena, Germany) on micrographs taken with the AxioImager.Z1 fluorescence microscope (Zeiss, Jena, Germany) at 10× and 20× magnification for mouse and human samples, respectively. At least 4 regions of interest were taken at 10× magnification, and 8 at 20× magnification. All micrographs were taken with the same exposure time at an excitation wavelength of 450–490 nm for green fluorescence (objective 10×: exposure time 840 ms; objective 20×: exposure time 100 ms) for all lectins. Using the software, the urothelium was then outlined on each of the micrographs, and densitometric values of fluorescence obtained in the selected area were transformed to grey values. The grey values of F.I. measured in arbitrary units (a.u.) were used for subsequent quantification. First, the average F.I. value of each micrograph was calculated, followed by calculation of gray values per 10,000 μm². Average values of F.I. for each human and mouse sample were then used for statistical analysis. 

**Figure 1:**
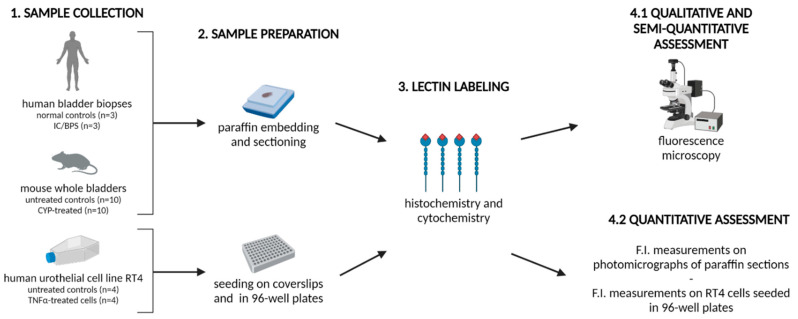
Schematic illustration of experimental design. The illustration was designed using Biorender.com (accessed on 16 March 2022). F.I., fluorescence intensity.

To quantitatively evaluate lectin binding in cell cultures, RT4 cells seeded in 96-well plates were first fixed with 10% buffered formalin for 15 min at RT, washed in PBS and blocked in 1% BSA in PBS for 1 h at RT. Cells were then incubated in a suspension of each FITC-conjugated lectin for 1 h at RT (Table 1). After the final wash, 200 µL of PBS was added to each well, and F.I. was measured at an excitation wavelength of 490 nm and an emission wavelength of 525 nm using a microplate reader (Safire; Tecan, Mannedorf, Switzerland). The F.I. of each lectin was measured in triplicate in four independent experiments (treated vs. untreated). The final F.I. of the sample was calculated by subtracting the mean F.I. of the background (unstained control) from the mean F.I. of the sample. A summary of experimental design is illustrated in Figure 1.

### 2.7. Statistical Analysis

F.I. values of urothelium-bound lectins were analyzed using GraphPad Prism version 6.01. Mean values of F.I. of each selected lectin presented as a.u. per 10,000 μm² were compared between CYP-treated and control mice, as well as normal human urothelium versus the urothelium of IC/BPS patients, using unpaired Student’s *t*-test. For in vitro data, the same test was used for the comparison of mean F.I. values expressed in a.u. between untreated and TNFα-treated RT4 cells. *p* values of <0.05 were considered statistically significant. Data are presented as mean ± SD for each independent in vitro experiment (*n* = 4 per group), animal (*n* = 10 per group), and human sample (*n* = 3 per group).

## 3. Results

### 3.1. Four of the Selected Lectins Bind to Human Urothelial Cells and IC/BPS Models

The results of lectin binding assessment revealed that only 4 of 10 selected lectins (ConA, DSL, Jacalin, and WGA) showed specific binding to normal and IC/BPS human urothelium with no semi-quantitative differences between normal and pathologic urothelium (Table 2). 

In contrast, we observed specific binding of 8 (ACA, ConA, DBA, DSL, Jacalin, SBA, UEA, and WGA) out of 10 lectins in mouse urothelium. However, the F.I. was weaker than in human tissue, which might be attributed to species differences. For three of the tested lectins (Jacalin, SBA, and UEA), we observed prominent differences in the binding to the urothelium of control versus CYP-treated mice. Specifically, the binding of these three lectins was completely absent in CYP-treated urothelium compared to weak binding observed in control urothelium. There were no significant differences in binding of other five lectins between the control and CYP-treated urothelium (Table 2). 

In in vitro experiments, measurements of F.I. showed binding of 6 (ConA, DSL, Jacalin, RCA, UEA, and WGA) out of the 10 lectins to RT4 cells (Table 2). There were no differences in lectin binding between TNFα-treated cells and untreated controls. We expected the lectin binding pattern in RT4 cells to be the same as in human urothelium, but in contrast to human tissue samples, an intense UEA and RCA reaction was observed in RT4 urothelial cells. This may be due to alterations in glycosylation during oncogenic transformation, since the RT4 cell line is derived from a non-muscle invasive bladder cancer [40,41].

Based on aforementioned results, we selected four lectins (ConA, DSL, Jacalin, and WGA) for differential analysis of glycosylation patterns in human urothelium, mouse urothelium, and RT4 cells.

### 3.2. Glycosylation Patterns of Human Urothelium Are Changed in IC/BPS

**Figure 2:**
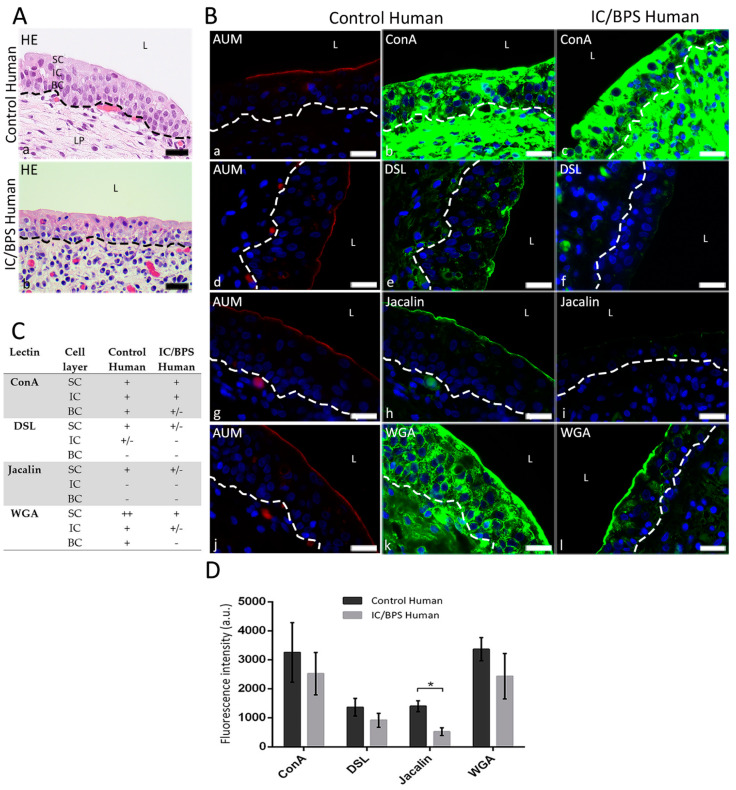
Lectin binding to normal and IC/BPS human urothelium. (**A**) Representative photomicrographs of HE-stained paraffin sections (**a**,**b**). (**B**) Representative photomicrographs of AUM immunolabeling (red fluorescence) and lectin fluorescence (green) in normal (**a**,**b**,**d**,**e**,**g**,**h**,**j**,**k**) and IC/BPS human urothelium (**c**,**f**,**i**,**l**). ConA labeling is very heterogeneous, ranging from very intense to weak in urothelial cells of control tissue (**b**), whereas in IC/BPS urothelium, BC show exclusively weak labeling (**c**). DSL labeling is present in the cytoplasm of individual IC and in the cytoplasm and apical surface of SC of normal urothelium (**e**), whereas in IC/BPS tissue, the labeling of DSL is almost absent except the very weak labeling of the apical surface of SC (**f**). Jacalin specifically labels the apical surface of SC, (**h**), whereas the labeling is very weak in the whole IC/BPS urothelium (**i**). The WGA labeling extends homogeneously throughout the urothelium in normal tissue (**k**), whereas the labeling in IC/BPS tissue gradually increases from weak labeling in BC to the very intense labeling of apical surface and cytoplasm of SC (**l**). (**C**) Semi-quantitative analysis of lectin binding to urothelial cells per cell layer (SC, superficial cells; IC, intermediate cells; BC, basal cells). Binding of lectins is scored as ++ (very intense), + (intense), +/− (weak) and − (very weak). (**D**) Fluorescence intensities of lectin labeling of human urothelium presented as a.u./10,000 μm². Means ± SD of three samples per group are shown. Student’s *t*-test was used to compare fluorescence intensities of each lectin between normal and IC/BPS urothelium. * *p* < 0.05. SC, superficial cells; IC, intermediate cells; BC, basal cells; L, lumen of the bladder; LP, lamina propria; dashed line indicates basal lamina. Nuclei are stained with Höechst stain (blue fluorescence). Scale bars: 20 μm.

In human urothelium, the predominant site of lectin binding were SC. In normal urothelium, ConA and WGA labeled the cytoplasm of SC, whereas DSL and Jacalin bound mostly to the apical surface of SC (Figure 2B(b,e,h,k),C). The labeling of deeper cell layers of the urothelium (IC and BC) was heterogeneous for ConA and DSL, while WGA and Jacalin labeling was homogeneously intense or very weak, respectively.

In the samples of IC/BPS patients, the Jacalin and DSL staining was very weak or almost completely absent in all urothelial cells (Figure 2B(f,i),C), whereas ConA maintained a strong staining pattern in SC and IC, but not in the BC (Figure 2B(c)). The WGA labeling strongly differed in the IC/BPS patient’s urothelium compared to normal human urothelium. It changed from intense labeling of all urothelial cells in control urothelium to gradually increased labeling from the BC to SC in IC/BPS urothelium (Figure 2B(l)).

The results of F.I. measurements showed intense staining of WGA and ConA in both normal human and IC/BPS human urothelium, while DSL and Jacalin staining intensities were evidently weaker. F.I. of bound Jacalin was significantly lower in the urothelium of IC/BPS bladders compared to normal human bladders (Figure 2D; $F.I._{\mathrm{normal}}$ = 1396.0 ± 186.6 a.u., $F.I._{\mathrm{IC}/\mathrm{BPS}}\;$= 522.9 ± 131.4 a.u., *p* = 0.0187). The other three analyzed lectins, ConA ($F.I._{\mathrm{normal}\;}$= 3253.0 ± 1028.0 a.u., $F.I._{\mathrm{IC}/\mathrm{BPS}}\;$= 2521.0 ± 725.7 a.u.), DSL $(F.I._{\mathrm{normal}}\;$= 1361.0 ± 302.3 a.u.$,\;F.I._{\mathrm{IC}/\mathrm{BPS}}\;$= 916.2 ± 237.6 a.u), and WGA ($F.I._{\mathrm{normal}}\;$= 3363.0 ± 399.8 a.u., $F.I._{\mathrm{IC}/\mathrm{BPS}}\;$= 2435.0 ± 782.4 a.u.), showed a similar trend of decreased F.I. in the IC/BPS group compared to control group, however the difference was not statistically significant. The lack of significance could be due to the small number of samples and the lectin-binding heterogeneity between them (large SD).

### 3.3. Glycosylation Is Significantly Diminished in Mouse Urothelium after CYP-Treatment

**Figure 3:**
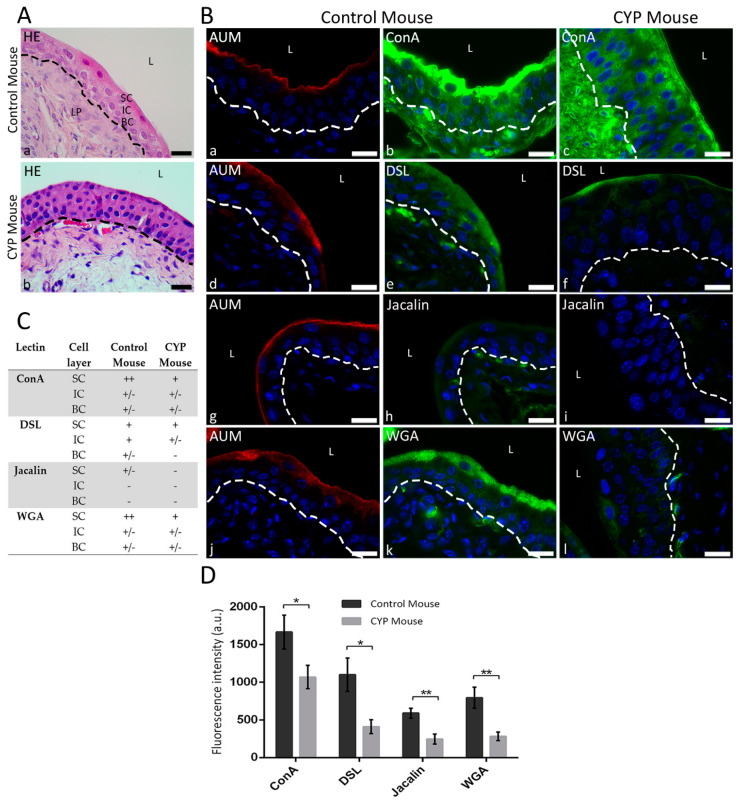
Lectin binding to control and CYP-treated mouse urothelium. (**A**) Representative photomicrographs of HE-stained paraffin sections (**a**,**b**). (**B**) Representative photomicrographs of AUM immunolabeling (red fluorescence) and lectin fluorescence (green) in normal (**a**,**b**,**d**,**e**,**g**,**h**,**j**,**k**) and CYP-treated mouse urothelium (**c**,**f**,**i**,**l**). ConA labeling is present in all cells of control and CYP-treated urothelium with very intense labeling in the cytoplasm of SC (**b**,**c**). DSL binds to all cell layers of control urothelium, whereas in CYP-treated tissue the labeling is limited to the apical surface of SC (**e**,**f**). Jacalin binds weakly to the cytoplasm of SC in control urothelium, whereas in the CYP-treated urothelium the Jacalin binding is almost completely absent (**h**,**i**). Very intense WGA binding is present in the cytoplasm of SC, while the binding is weak in IC and BC of normal mouse urothelium. After treatment with CYP, fluorescence intensity is decreased in SC layer, while IC and BC continue to show weak WGA binding (**k**,**l**). (**C**) Semi-quantitative analysis of lectin binding to urothelial cells per cell layer (SC, superficial cells; IC, intermediate cells; BC, basal cells). Binding of lectins is scored as ++ (very intense), + (intense), +/− (weak) and − (very weak). (**D**) Fluorescence intensities of lectin labeling of mouse urothelium presented as a.u./10,000 μm². Means ± SD of 10 samples per group are shown. Normal and CYP-treated groups are compared for each lectin. There is a significant reduction in lectin fluorescence in CYP samples. The reduction is statistically significant for all lectins (* *p* < 0.05 and ** *p* < 0.01). SC, superficial cells; IC, intermediate cells; BC, basal cells; L, lumen of bladder; LP, lamina propria; dashed line indicates basal lamina. Nuclei are stained with Höechst stain (blue fluorescence). Scale bars: 20 μm.

In bladders of control mice, the cytoplasm of SC was strongly labeled with ConA, followed by DSL and WGA (Figure 3B(b,e,k),C), whereas IC and BC were less intensely stained with these three lectins. In the urothelium of CYP-treated mice, the intensity of ConA and WGA labeling was evidently reduced especially in the cytoplasm of SC, while DSL labeling was found exclusively at the apical surface of SC (Figure 3B(c,f,l),C). The binding of Jacalin was very weak in all urothelial cells of control bladders, and it almost completely disappeared in the urothelium of CYP-treated animals (Figure 3B(h,i),C).

The results of F.I. measurements revealed that the labeling of all four analyzed lectins was significantly reduced in CYP-treated urothelium compared to controls (Figure 3D; *ConA*: $F.I._{\mathrm{ctrl}}\;$= 1665.0 ± 224.5 a.u., $F.I._{\mathrm{CYP}}\;$= 1067.0 ± 155.4 a.u., *p* = 0.0419; *DSL*: $F.I._{\mathrm{ctrl}}\;$= 1099.0 ± 222.0 a.u., $F.I._{\mathrm{CYP}}\;$= 411.2 ± 90.84 a.u., *p* = 0.0102; *Jacalin*: $F.I._{\mathrm{ctrl}}\;$= 588.9 ± 65.54 a.u., $F.I._{\mathrm{CYP}}\;$= 246.6 ± 67.09 a.u., *p* = 0.0018; *WGA*: $F.I._{\mathrm{ctrl}}\;$= 792.5 ± 140.3 a.u., $F.I._{\mathrm{CYP}}\;$= 282.3 ± 57.04 a.u., *p* = 0.0034). 

### 3.4. Glycosylation Patterns Remain Unchanged in TNFα-Treated RT4 Cells

**Figure 4:**
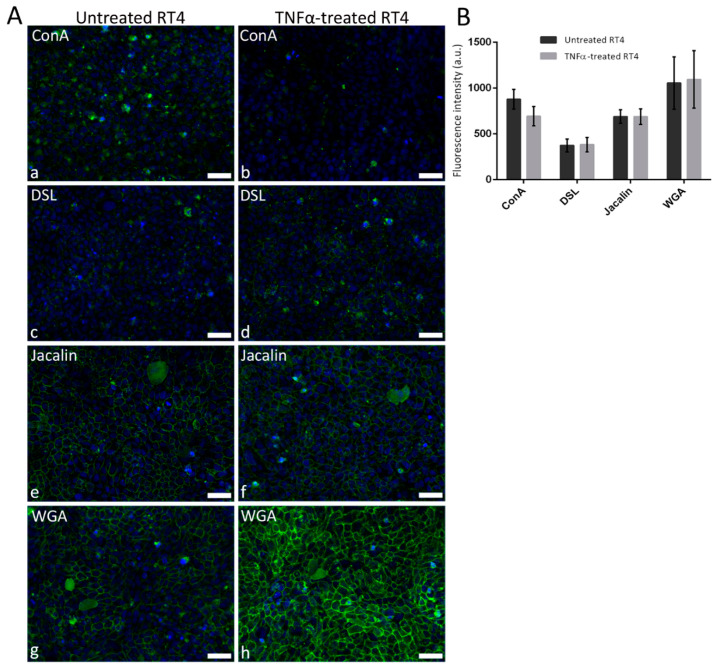
Lectin binding to control and TNFα-treated human urothelial RT4 cell line. (**A**) Representative micrographs of lectin fluorescence (green) in untreated controls (**a**,**c**,**e**,**g**) and TNFα-treated cells (**b**,**d**,**f**,**h**). Note that ConA and DSL label the cytoplasm (**a**–**d**), whereas Jacalin and WGA show a higher affinity for the plasma membrane. (**B**) Absolute values of fluorescence intensities in the RT4 cell line. Means ± SD of four independent experiments are shown. Nuclei are stained with DAPI (blue fluorescence). Scale bars: 50 μm.

In the RT4 urothelial cells (control and TNFα-treated), either the cytoplasm (for ConA and DSL) or the plasma membrane (for Jacalin and WGA) was the predominant lectin binding site (Figure 4A). There were no prominent differences in lectin binding between untreated and TNFα-treated RT4 cells.

The results of F.I. measurements indicated the most prominent staining with WGA and the weakest with DSL (Figure 4B). F.I. values of the selected lectins showed no significant changes between untreated and TNFα-treated cells (Figure 4B; *ConA*: $F.I._{\mathrm{ctrl}}$ = 877.7 ± 107.3 a.u., $F.I._{\mathrm{TNF}\alpha }$= 693.7 ± 104.4 a.u.; *DSL*: $F.I._{\mathrm{ctrl}}$ = 374.2 ± 69.6 a.u., $F.I._{\mathrm{TNF}\alpha }$ = 382.6 ± 79.75 a.u.; *Jacalin*: $F.I._{\mathrm{ctrl}}$ = 689.9 ± 72.9 a.u., $F.I._{\mathrm{TNF}\alpha }$ = 688.6 ± 84.34 a.u.; *WGA*:$\;F.I._{\mathrm{ctrl}}$ = 1056.0 ± 286.2 a.u., $F.I._{\mathrm{TNF}\alpha }$ = 1095.0 ± 313.5 a.u.).

## 4. Discussion

The aim of our study was to investigate the differences in glycosylation patterns between normal and pathologically altered urothelium of patients with IC/BPS using FITC-conjugated plant lectins in order to find out whether lectins could serve as potential new biomarkers for the pathohistological diagnosis of IC/BPS. In addition to human samples, two of the most widely used experimental models for preclinical research of IC/BPS were included in the study, namely CYP-induced mouse model of chronic bladder inflammation and TNFα-treated human urothelial RT4 cells. We evaluated the binding of 10 lectins in human urothelium, mouse urothelium, and RT4 cells. Among them, ConA, DSL, Jacalin, and WGA exhibited specific binding to the urothelial cells in all three types of analyzed samples.

It is generally accepted that the luminal surface of the urothelium is lined with a glycocalyx, particularly non-sulfated hyaluronic acid and sulfated GAG (chondroitin sulfate, heparan sulfate, keratan sulfate, and dermatan sulfate), which contribute to the urothelial barrier function and reduce the adhesion of uropathogenic bacteria. Moreover, loss of GAG is thought to be one of the major factors in the development of a ‘‘leaky bladder’’ in IC/BPS [8]. Not surprisingly, replenishment of GAG is one of the American Urological Association (AUA)-approved therapeutic approaches for IC/BPS [9], which has also been reported to be effective in various in vivo and in vitro models of IC/BPS [48]. We therefore expected to detect altered glycosylation patterns in the urothelium of IC/BPS patients and experimental models compared to normal tissue, which was confirmed by different approaches used in the present study.

In the pioneering studies of urothelial glycosylation, the urothelium was examined using various histochemical techniques, such as Alcian blue, colloidal iron, and PAS staining, where a positive staining was found in the apical surface of SC [49,50], which was attributed to the glycocalyx. Interestingly, more recent studies have demonstrated that although the GAG layer is visible on histological bladder specimens, very little GAG can be isolated biochemically. Several studies indicate that the urothelium contains relatively low concentrations of GAG and that GAG are mainly found in the lamina propria and smooth muscle layer of the bladder [7,10]. For example, Hurst and colleagues were able to isolate heparan sulfate and chondroitin sulfate in only moderate concentrations from rat bladders, and estimated the GAG density in the bladder luminal surface to be one molecule per 50 nm² [7]. In contrast, there is evidence for higher glycoprotein concentrations in rabbit and calf bladders, and to some extent in normal human bladders as well [10,11,50]. Our results are partly consistent with the aforementioned data since lectin binding to sugar residues at apical surface of SC was observed. However, we were not able to detect a distinct glycocalyx layer. Furthermore, we also detected lectin binding in the cytoplasm of urothelial cells, which could be expected, since the majority of protein glycosylation occurs in the Golgi apparatus. On the other hand, lectin binding in the cytoplasm in our samples can also be attributed to the selected CLIH protocol (addition of lectins directly to tissue sections), in contrast to some of the other studies where lectins were added ex vivo or en bloc, and the lectin reaction was restricted only to the apical plasma membrane of SC [38,51].

Similar to the measurements of fluorescence intensity of urothelial cells in normal human bladders obtained by Ward and coworkers [29], our examination of glycosylation patterns in human urothelium demonstrated that the apical surface of SC is the primary binding site for studied lectins. High F.I. values of WGA and ConA were detected in both normal and IC/BPS urothelium, indicating high contents of syaliated GlcNAc and N-linked mannose in this tissue. The trend of reduced F.I. in pathologic versus normal urothelium was evident for all four studied lectins, with the reduction being statistically significant only for Jacalin. Semi-quantitative evaluation of lectin binding also showed a distinct change in the binding pattern of WGA in IC/BPS urothelium in comparison to normal urothelium. We therefore suggest that a combination of quantitative analysis of Jacalin binding and qualitative assessment of WGA binding could be used as additional methods for histopathological diagnosis of IC/BPS in patients. To our knowledge, this is the first study to combine a detailed qualitative and quantitative analysis of lectin binding in the urinary bladders of patients with IC/BPS.

Our results of lectin binding analysis obtained in the urothelium of control mice are partly in accordance with the research conducted by Zupančič and coworkers on ex vivo normal mouse urothelium, where strong WGA binding, weak Jacalin binding, and weakest DSL binding were shown [38]. The results of our study show a significant reduction of lectin binding in the urothelium of CYP-treated animals compared to controls, especially in the cytoplasm of SC. The major lectin binding site in mouse urothelium were SC, with ConA binding being the most intense, indicating high concentrations of N-linked mannose. Jacalin bound weakly to the apical surface of SC, suggesting low concentrations of Core1- and Core2-linked GalNAc in the urothelium of mice. As expected, the F.I. of the selected lectins was also evidently reduced in the urothelium of CYP-treated mice compared to controls. To summarize, our results show strong similarity in glycopatterns of human IC/BPS urothelium and CYP-treated mouse urothelium, proving that the presented in vivo model is suitable and reliable for studying the glycobiology of IC/BPS.

Glycosylation patterns of normal human urothelium are rarely quantitatively analyzed due to difficulties in obtaining the specimens from healthy individuals. However, there is a large number of studies available regarding the glycosylation of different human urothelial cell lines [39,40,41]. Interestingly, our results obtained on the RT4 cell line are in complete contrast to the glycopattern analysis of human non-muscle-invasive bladder cancer cells KK47 performed by Yang and colleagues, which showed 5-fold higher levels of N-linked GalNAc glycoconjugates detected with DSL compared to any other glycans detected with ConA, WGA, or Jacalin [40]. These differences could be due to the different methodological approaches used in the current study and the Yang’s study, in which the extraction of total protein from cells and the use of lectin microarrays for quantitative analysis were performed. The results of our study surprisingly showed minimal changes in F.I. and lectin binding distribution of all analyzed lectins in RT4 cells after TNFα treatment. ConA and DSL bound preferably to the cytoplasm, while Jacalin and WGA bound to the plasma membrane in both treated and untreated RT4 cells. Mean values of F.I. for WGA and ConA were the highest, showing high levels of syaliated GlcNAc and N-linked mannose in RT4 cells, with no significant effect of TNFα treatment on change in F.I. values. We can conclude that some similarities can be found between the glycopatterns of RT4 cells and normal human urothelium, namely the distribution of ConA and Jacalin, together with high F.I. values of WGA and ConA, however there are even fewer similarities present between the TNFα-treated RT4 cells and the urothelium of patients with IC/BPS. We, therefore, assume that the cancerous origin of the RT4 cell line and the non-complexity of a cell culture might hinder the use of this in vitro model for the research of IC/BPS glycobiology.

The ability of lectins to recognize different sugar residues expressed on the cell surface could have a great therapeutic value. Effective intravesical drug application remains extremely challenging due to low urothelial permeability and periodical voiding, resulting in fast drug clearance and the necessity for repeated catheterization. Different biomaterials for drug encapsulation are already being explored, as well as drug delivery systems that could increase specificity of active substance release and increase drug dwelling time in the bladder [52]. Therefore, the change of glycosylation patterns in urothelium under different pathological conditions could be exploited for lectin-assisted targeted drug delivery. Neutsch and coworkers have already shown the great binding capacity and internalization of WGA-bioconjugates to different human urothelial cell lines [53,54,55]. These results were later confirmed by Apfelthaler et al., who designed a bioconjugate of polymeric backbone with high drug loading capacity, a fluorescent molecule enabling tracking, and WGA for specific adhesion and internalization of the drug delivery system to human bladder carcinoma cells [56]. The results of our study indicate an increase in WGA binding to SC in the urothelium of patients with IC/BPS, which could be exploited for development of targeted lectin-assisted drug delivery for the treatment of IC/BPS in the future.

Taken together, lectins represent a very useful tool for studying glycosylation properties of different tissues and their changes under various pathological conditions. To our knowledge, we are the first to evaluate in detail the expression of glycoconjugates in IC/BPS human urothelium and to compare it with widely used experimental models of IC/BPS. Our results undoubtedly show a distinct change in the WGA binding pattern in the urothelium of IC/BPS patients, as well as a pronounced reduction in the intensity of Jacalin binding. We, therefore, propose WGA and Jacalin as potential auxiliary biomarkers in the confirmation of a histopathological diagnosis of IC/BPS. Our study also demonstrates that the resemblance in glycosylation patterns between IC/BPS human urothelial tissue and CYP-treated mouse urothelial tissue is sufficient for the use of this animal model in the investigation of IC/BPS glycobiology, as well as the discovery of potential new drug targets. Nevertheless, the results obtained in models of human pathologies should always be carefully interpreted before translation into a clinical setting.

## Figures and Tables

**Table 1:** The lectins used in the experiments. The preferred sugar specificity, preferred carbohydrate structure for lectin binding, manufacturers, and dilutions are listed. The optimal dilutions for each lectin were determined in preliminary experiments.

Lectin	Abbreviation	Preferred Monosaccharide Specificity (According to [42])	Preferred Carbohydrate Structure for Lectin Binding (According to [42])	Manufacturer and Dilution (Cell Culture/Tissue)
*Amaranthus caudatus* agglutinin	ACA	GlcNAc	Core1 and Core2	#21761009-1, Bioworld, Dublin, USA; 1:50/1:800
Concavalin A, (Jackbean lectin)	ConA	Man, Glc	N-linked Man	#FLK-2100, Vector Labs, Burlingame, USA; 1:300/1:400
*Dolichos biflorus* agglutini	DBA	GalNAc	GalNAcβ1-3GalNAc	#FLK-2100, Vector Labs, Burlingame, USA, 1:50/1:200
*Datura stramonium* lectin	DSL	GlcNAc	N-linked (Galβ1-4GlcNAc-)n	#FL-1181, Vector Labs, Burlingame, USA; 1:200/1:100
Jacalin	Jacalin	GalNAc	Core1 and Core2	#FL-1151, Vector Labs, Burlingame, USA; 1:400/1:1500
Peanut agglutinin	PNA	Gal	N-linked Galβ1-3GalNAc	#FLK-2100, Vector Labs, Burlingame, USA; 1:50/1:400
*Ricinus communis* agglutinin I	RCA I	Gal	LacNAcβ, GalNAcβ, Galβ, Lacβ	#FLK-2100, Vector Labs, Burlingame, USA; 1:200/1:200
Soybean agglutinin	SBA	GalNAc	O-linked GalNAcα1-3Gal	#FLK-2100, Vector Labs, Burlingame, USA; 1:50/1:400
*Ulex Europeaus* agglutinin I	UEA I	Fuc	Fucα1-2Galβ1-4GlcNAc	#FLK-2100, Vector Labs, Burlingame, USA; 1:400/1:400
Wheat Germ agglutinin	WGA	GlcNAc, syaliated	GlcNAc(β1-4GlcNAc)n	#FLK-2100, Vector Labs, Burlingame, USA; 1:800/1:1000

**Table 2:** The results of semi-quantitative assessment of lectin binding in normal and IC/BPS human urothelium, control and CYP-treated mouse urothelium, and control and TNFα-treated RT4 cells. Legend: ++ (very intense), + (intense), +/− (weak), or − (very weak).

Lectin	Human Urothelium		Mouse Urothelium		RT4 Cell Line	
	Control	IC/BPS	Control	CYP	Control	TNFα
ACA	−	−	+/−	+/−	−	−
ConA	++	++	+	+	+	+
DBA	−	−	+/−	+/−	−	−
DSL	+	+	+	+	+/−	+/−
Jacalin	+	+	+/−	−	++	++
PNA	−	−	−	−	−	−
RCA I	−	−	−	−	+	+
SBA	−	−	+/−	−	−	−
UEA I	−	−	+/−	−	+	+
WGA	++	++	+	+	++	++

## Data Availability

All the collected and analyzed data is presented and discussed in the article.

## Abbreviations

a.u.	arbitrary units
AUA	American Urological Association
AUM	asymmetric unit membrane
BC	basal cells
BSA	bovine serum albumin
CLIH	combined lectin- and immuno-histochemistry
CRD	carbohydrate recognition domain
CYP	cyclophosphamide
ESSIC	International society for the study of BPS
F.I.	fluorescence intensity
FITC	fluorescein isothiocyanate
Fuc	fucose
GAG	glycosaminoglycans
Gal	galactose
GalNAc	*N*-acetylgalactosamine
Glc	glucose
GlcNAc	*N*-acetylglucosamine
IBD	inflammatory bowel disease
IC	intermediate cells
IC/BPS	interstitial cystitis/bladder pain syndrome
Man	mannose
PAS	periodic acid-Schiff
PBS	phosphate buffered saline
RT4	human urothelial carcinoma cell line
SC	superficial cells
TNFα	tumor necrosis factor alpha
